# Association of raloxifene and tamoxifen therapy with cognitive performance, odds of mild cognitive impairment, and brain MRI markers of neurodegeneration

**DOI:** 10.1002/cam4.5175

**Published:** 2022-08-30

**Authors:** Firat Kara, Christine M. Lohse, Anna M. Castillo, Nirubol Tosakulwong, Timothy G. Lesnick, Clifford R. Jack, Ronald C. Petersen, Janet E. Olson, Fergus J. Couch, Kathryn J. Ruddy, Kejal Kantarci, Michelle M. Mielke

**Affiliations:** ^1^ Department of Radiology Mayo Clinic Rochester Minnesota USA; ^2^ Department of Quantitative Health Sciences Mayo Clinic Rochester Minnesota USA; ^3^ Department of Neurology Mayo Clinic Rochester Minnesota USA; ^4^ Health Sciences Research Mayo Clinic Rochester Minnesota USA; ^5^ Department of Laboratory Medicine and Pathology Mayo Clinic Rochester Minnesota USA; ^6^ Department of Oncology Mayo Clinic Rochester Minnesota USA; ^7^ Department of Epidemiology and Prevention Wake Forest University School of Medicine Winston‐Salem North Carolina USA

**Keywords:** breast cancer, cognitive performance, cross‐sectional, MCI, raloxifene, tamoxifen

## Abstract

The aim of this cross‐sectional study was to examine whether a history of selective estrogen receptor modifiers (SERMs), tamoxifen and raloxifene, use was associated with cognitive performance, odds of mild cognitive impairment (MCI), or magnetic resonance imaging (MRI) markers of neurodegeneration associated with Alzheimer's disease. We included women with prior history of breast cancer or no prior history of any cancer at enrollment in the Mayo Clinic Study of Aging (MCSA). This information was abstracted using the Rochester Epidemiology Project medical‐linkage system. Logistic regression was used to examine associations of SERMs with odds of MCI. Linear regression models were used to examine associations of SERMs with cognitive z‐scores (Memory, Executive Function, Language, Visuospatial Skills, Global Cognition), and MRI markers. Among 2840 women aged 50 and older in the MCSA, 151 had a history of breast cancer, and 42 (28%) of these had a history of tamoxifen treatment. A total of 2235 women had no prior history of any cancer, and 76 (3%) of these had a history of raloxifene use. No significant associations between tamoxifen use and cognition, or odds of MCI were observed among women with a history of breast cancer after adjusting for confounders. Similarly, raloxifene use was not significantly associated with cognition, or odds of MCI in women without a history of cancer after adjusting for confounders. We did not find significant associations between the use of either SERM and MRI markers. Use of tamoxifen or raloxifene was not significantly associated with cognition in postmenopausal women.

## Graphical abstract

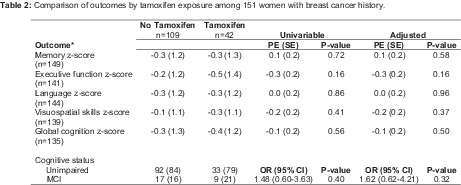

**Novelty and Impact**

There are multiple novel aspects to this study. First, we investigated associations of tamoxifen and raloxifene with global and domain‐specific cognition. Second, we studied associations of these medications with odds of mild cognitive impairment (MCI) and neuroimaging markers of neurodegeneration. Together, our results demonstrate that the use of these medications was not associated with global or domain‐specific cognition, odds of MCI, or structural MRI markers of neurodegeneration among women with and without a history of breast cancer.

## 1 INTRODUCTION

Breast cancer is the most prevalent malignancy in women,^1^ and two thirds of breast cancers are estrogen receptor positive (ER+). Elevated levels of sex hormones, such as estrogen, have been associated with an increased risk of breast cancer due to the proliferative effects of estrogen on mammary tumors.^2^ Hormone modifying therapies, such as the selective estrogen receptor modulators (SERMs) tamoxifen and raloxifene decrease the risk of breast cancer or recurrence rate by blocking the effects of estrogen in breast tissue.^3^ Raloxifene is also commonly used for prevention and treatment of osteoporosis in postmenopausal women. Although tamoxifen and raloxifene act as estrogen receptor antagonists in breast tissue, their action as estrogen agonists or antagonists in the human brain is less clear. Understanding the potential effects of tamoxifen and raloxifene on cognitive performance and risk of mild cognitive impairment (MCI),^4^ an intermediate clinical stage between normal aging and dementia, is of great importance.

Studies examining the effects of tamoxifen and raloxifene on cognitive decline and risk of MCI or Alzheimer's disease (AD) have reported mixed results. Some studies have reported that tamoxifen was associated with cognitive impairment.^5^, ^6^ Furthermore, some studies have reported that tamoxifen, raloxifene, or aromatase inhibitors (i.e., hormone therapy that lowers the estrogen production throughout the body if there is no ovarian production of estrogen) were associated with a lower prevalence of AD.^7^, ^8^ The discrepancy between results might be due to differences in study designs and study limitations. For example, some studies investigated the effects of hormone‐modulating therapies (HMTs: SERMs and aromatase inhibitors) on cognitive performance without distinguishing between SERMs and aromatase inhibitors.^9^ Some studies only compared a group of women without a personal history of breast cancer who did not use any HMT with women who had breast cancer to study the effect of HMTs on cognitive performance.^10^ This is problematic because aspects of having breast cancer (e.g., chemotherapy, stress) may also contribute to cognitive decline. Lastly, several studies used global cognitive tests or diagnostic codes for dementia and did not examine specific cognitive domains or adjudicate a clinical diagnosis of dementia.

Information acquired from neuroimaging methods such as magnetic resonance imaging (MRI) can be complementary to cognitive assessment and contribute to better understanding of the brain changes due to tamoxifen and raloxifene, even among those who do not yet have cognitive impairment. One study examined the effect of tamoxifen on hippocampal volume.^11^ Findings of this study suggest that tamoxifen may alter the brain structure. However, the result of study was confounded by a relatively small sample size. To our knowledge, earlier studies have not examined the association between SERMs with temporal lobe cortical thickness, which is characteristic of AD and related disorders. Moreover, the association between raloxifene use and hippocampus volume in women without personal history of breast cancer has not been evaluated. Both hippocampus volume and temporal lobe cortical thickness are well‐validated biomarkers to detect progression in MCI and neurodegeneration associated with AD.^12^, ^13^

In this study, we analyzed the data from the population‐based Mayo Clinic Study of Aging (MCSA), which includes comprehensive neuropsychological testing and MRI data for a subset of participants. We aimed to (1) evaluate the association of previous tamoxifen use with global and domain‐specific cognition and odds of MCI among women with a history of breast cancer and (2) evaluate the association of previous raloxifene use with global and domain‐specific cognition and odds of MCI among women without a history of breast cancer. Furthermore, among a subset of women with brain MRI, we examined associations of tamoxifen and raloxifene use with hippocampus volume and temporal lobe cortical thickness associated with AD.

## 2 MATERIALS AND METHODS

### 2.1 Study population

We used data from the MCSA, a prospective population‐based study of the prevalence and incidence of cognitive decline and MCI and of risk factors associated with them among a representative sample of individuals living in Olmsted County, Minnesota.^14^ The MCSA study population consisted of participants aged 50 and older. Olmsted County residents were enumerated using the Rochester Epidemiology Project (REP) medical records linkage system.^15^ Details of the MCSA design and participant recruitment have previously been provided.^14^ The baseline MCSA visit occurred between 2004 and 2020, and included a physician examination, an interview by a study coordinator, and neuropsychological testing by a psychometrist.^14^ The physician examination included a neurological examination and administration of the Short Test of Mental Status.^16^ Study coordinator interviews included participants' demographic information, assessments of depression and anxiety, and the participant and informant Clinical Dementia Rating scale.^17^ A subset of women also agreed to have a brain MRI. There were no significant differences between those with versus without MRI with regard to education, medical conditions, or the presence of *APOE* ε4 (ε4 allele of the Apolipoprotein E), but in both the tamoxifen and raloxifene comparisons, women who underwent MRI were younger than women who did not (*p* < 0.05).

**FIGURE 1:**
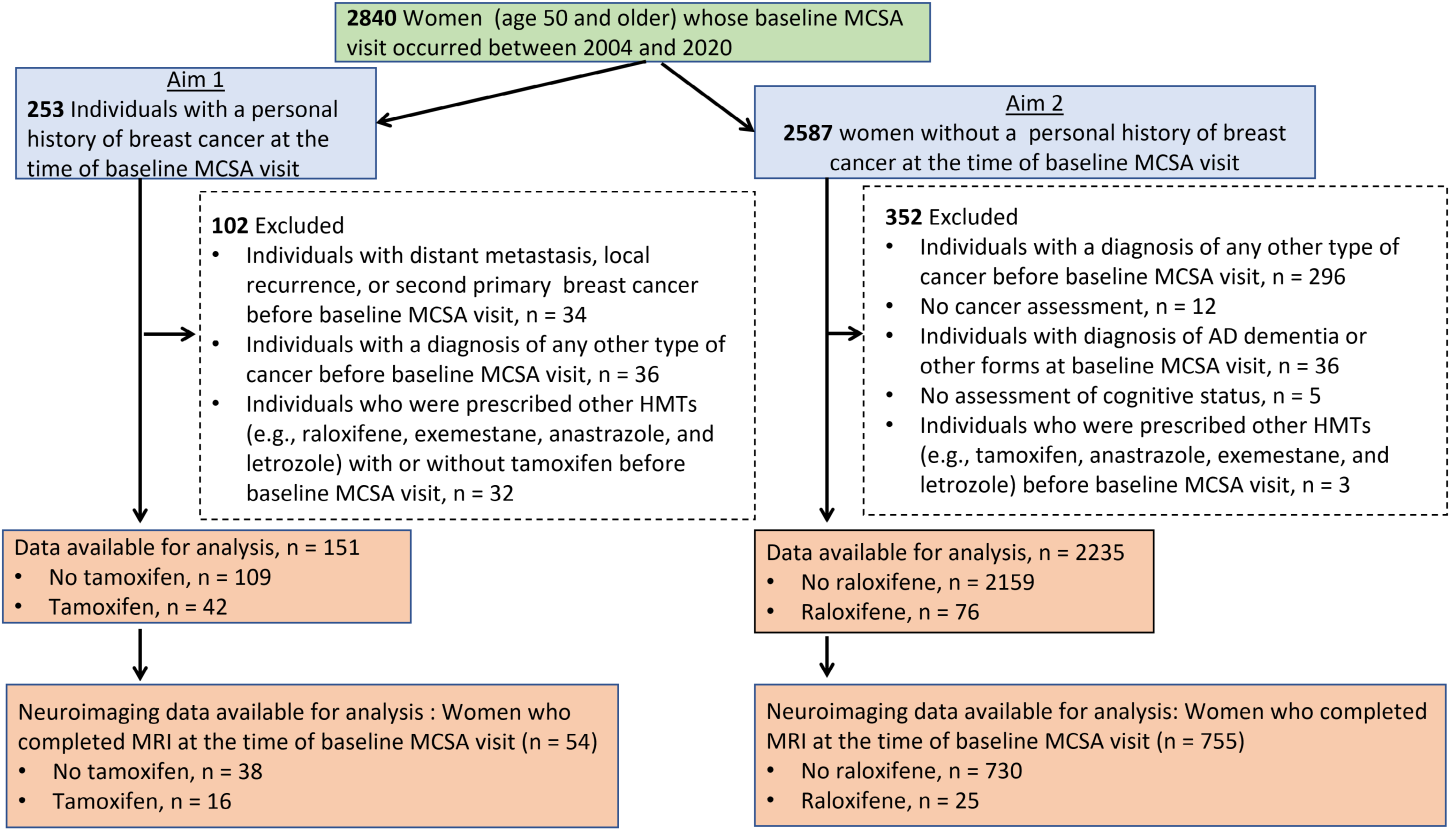
Flow chart of the study. MCSA, Mayo Clinic Study of Aging; N, number; MRI, magnetic resonance imaging

The flow chart of the study summarizing exclusion criteria is depicted in Figure 1. For Aim 1, in which we assessed the effects of tamoxifen, we included all women aged 50 and older enrolled in the MCSA who had been diagnosed with breast cancer before their baseline MCSA visit and who were not exposed to other HMTs including raloxifene, exemestane, anastrozole, and letrozole. Women with a history of metastatic breast cancer or other cancers before their baseline MCSA visit were excluded. For Aim 2, in which we assessed the effects of raloxifene, we identified all women aged 50 and older who had not been diagnosed with any cancer and had not been prescribed other HMTs (e.g., tamoxifen and aromatase inhibitors) prior to or at their baseline MCSA. In both aims, we also investigated associations of tamoxifen and raloxifene use with structural MRI measures of neurodegeneration related with AD.

A diagnosis of breast cancer, distant metastasis status, history of other types of cancers, use of HMTs, and other therapies were abstracted using the Rochester Epidemiology Project (REP) medical records linkage system.^15^ Nurses abstracted the medical records of all women enrolled in the MCSA and confirmed all cancer diagnoses. The following breast cancer variables were collected: date of diagnosis (including recurrence, surgery, and treatment type), radiation, chemotherapy, hormonal treatment, breast cancer hormone receptor status (e.g., positive, or negative for ER, progesterone receptor, and human epidermal growth factor receptor 2), and TNM (i.e., cancer staging system which stands for tumor, nodes, and metastases) classifications. “No tamoxifen or raloxifene” was defined as no history of a prescription before the baseline MCSA visit. This study was approved by the Mayo Clinic and Olmsted Medical Center Institutional Review Boards and written informed consent was obtained prior to participation. Data are available upon reasonable request (https://www.mayo.edu/research/centers‐programs/alzheimers‐disease‐research‐center/research‐activities/mayo‐clinic‐study‐aging/for‐researchers/data‐sharing‐resources).

### 2.2 Baseline cognitive testing and assessment of mild cognitive impairment

All tests were administered by experienced psychometrists and supervised by board‐certified clinical neuropsychologists. Four cognitive domains were assessed to evaluate cognitive performance by a widely used, well‐standardized battery of nine neuropsychological tests: (1) memory (Auditory Verbal Learning Test Delayed Recall Trial,^18^ Logical Memory subtest and Visual Reproduction subtest of the Wechsler Memory Scale‐Revised^19^); (2) attention and executive function (Trail Making Test,^20^ and Digit Symbol subtest of the Wechsler Adult Intelligence Scale‐Revised^21^); (3) language (Boston Naming Test^22^ and Category Fluency^23^); and (4) visuospatial (Picture Completion subtest and Block Design subtest of the WAIS‐R^21^). Raw scores from four cognitive domain tests were converted to *z*‐scores using means and standard deviations (SDs) from the subset of women in each aim classified as cognitively unimpaired. The four cognitive domain‐specific *z*‐scores were averaged to obtain a global cognition score that was converted to a *z*‐score.

Cognitive status was classified as cognitively unimpaired or MCI at the MCSA baseline visit. For each participant, raw scores in a cognitive domain were age‐adjusted and scaled using normative data from the Mayo's Older American Normative Studies.^24^ Participants with scores around 1.0 SD below the age‐specific mean in the general population were considered for possible cognitive impairment. The final clinical diagnosis about MCI was based on a consensus agreement among the physician, study coordinator, and neuropsychologist who interpreted cognitive data for each participant after considering variables such as education, prior occupation, visual or hearing deficits, and reviewing all other participant information using published criteria.^14^, ^25^ The diagnosis of dementia and AD was based on published criteria.^26^ Participants who did not meet criteria for MCI or dementia were classified as cognitively unimpaired. The clinical evaluations were performed without using information from neuroimaging or other biomarkers acquired during the study.

### 2.3 Structural MRI

Participants underwent MRI scans at baseline at 3 Tesla using an 8‐channel phased array coil (GE Healthcare, Waukesha, WI). A three‐dimensional, high‐resolution magnetization‐prepared rapid acquisition gradient echo was performed for hippocampal volume and cortical thickness measurements.^27^ Parameters were sagittal plane, repetition time/echo time/inversion time, 2300/3/900 ms; flip angle 8°, 26 cm; field of view (FOV); 256 × 256 in‐plane matrix with a phase FOV of 0.94 and slice thickness of 1.2 mm. All images were corrected for gradient non‐linearity and intensity non‐uniformity.^28^ Hippocampal volumes were measured using FreeSurfer software version 5.3 on each T1‐weighted scan. Regional cortical thickness measurements were estimated using FreeSurfer 5.3 software with default settings.^29^ AD‐related temporal lobe cortical thickness measure was composed of the following individual cortical thickness regions of interest: entorhinal, inferior temporal, middle temporal, and fusiform.^28^ Atrophy in this temporal lobe meta region of interest is characteristic of typical AD and related disorders such as LATE disease.^12^, ^30^ Total intracranial volumes (TIVs) were measured using an algorithm developed in‐house. Hippocampal volume was adjusted for TIV.

### 2.4 Assessment of covariates

Demographic variables were obtained by self‐report. Participants' height (cm) and weight (kg) were measured during the in‐clinic exam and used to calculate body mass index (BMI) (kg/m^2^). The REP medical record linkage system was used to abstract information on medical conditions (e.g., diabetes, hypertension, diabetes, stroke, and heart disease), menopausal status at the baseline visit, age at menopause, and use of menopausal hormone therapy (MHT). APOE genotyping was performed from a blood sample drawn at clinic examination. Depressive symptoms were assessed using the Beck Depression Inventory‐II; a score of 13 was considered clinical depression.^31^

### 2.5 Statistical methods

Continuous features were summarized with means and standard deviations (SDs) and categorical features were summarized with frequencies and percentages. Comparisons of covariates and outcomes between women with and without SERMs were evaluated using two‐sample t tests for continuous features, Wilcoxon rank sum tests for ordinal features, and chi‐square or Fisher exact tests for categorical features. Unless it was indicated otherwise, logistic (to examine odds of MCI) and linear regression (to examine continuous cognitive outcomes) models were adjusted for confounders that were associated with the risk of MCI including age, education, APOE genotype, BMI, stroke, and heart disease.^32^ For analysis of data from women with breast cancer, stroke was not included in the multivariable adjustment for the domain and the global cognitive z‐scores because only four women had a history of stroke. The only covariate included in the multivariable adjustment for MCI was age because the limited number of women with MCI (*n* = 29) could not support more extensive multivariable modeling. Statistical analyses were performed using SAS version 9.4 (SAS Institute; Cary, NC) and R statistical software version 3.6.2 (R project.org). All tests were two‐sided and *p*‐values <0.05 were considered statistically significant.

## 3 RESULTS

### 3.1 Tamoxifen use and cognition

Among the 2840 women aged 50 and older at enrollment in the MCSA, 151 had a history of breast cancer and were included in this analysis (Table 1). The mean age (SDs) of the women was 77 (8), and the mean years of education was 14 (2). Of the 151 women with a history of breast cancer, 42 (28%) used tamoxifen and 109 (72%) did not. The only significant differences between those who did and did not take tamoxifen were estrogen receptor status and TNM classification (*p* < 0.05). Most women used tamoxifen before they enrolled the MCSA, but there were 10 women (6.6%) who were using tamoxifen at the time of enrollment.

**TABLE 1:** Characteristics of participants with personal history of breast cancer

	No tamoxifen mean (SD)/*n*(%) *n* = 109	Tamoxifen mean (SD)/*n*(%) *n* = 42	*p*‐value
**Characteristic** ^a^			
Age in years	77 (9)	77 (8)	0.91
Race (*n* = 150)			
All others	1 (1)	1 (2)	NE
White	107 (99)	41 (98)	
Ethnicity (*n* = 149)			
Hispanic or Latino	0	0	NE
Not Hispanic or Latino	107 (100)	42 (100)	
Years of education	14 (2)	14 (2)	0.81
Years of education			
≤12	44 (40)	18 (43)	0.78
>12	65 (60)	24 (57)	
BMI in kg/m^2^ (*n* = 145)	27.5 (5.0)	28.2 (5.1)	0.45
BMI in kg/m^2^ (*n* = 145)			
<18.5	2 (2)	0	0.50
18.5–24.9	36 (34)	12 (30)	
25.0–29.9	36 (34)	15 (38)	
≥30.0	31 (30)	13 (33)	
Comorbidities			
Diabetes	17 (16)	11 (26)	0.13
Hypertension	81 (74)	32 (76)	0.81
Stroke	4 (4)	0	NE
Heart disease	36 (33)	12 (29)	0.60
BDI II (*n* = 144)			
<13	101 (97)	36 (90)	0.09
≥13	3 (3)	4 (10)	
APOE status (*n* = 145)			
All others	76 (73)	28 (68)	0.56
Any ε4	28 (27)	12 (32)	
Menopause	107 (98)	42 (100)	NE
Age at menopause in years (*n* = 138)	48 (5)	47 (7)	0.12
MHT use	48 (44)	14 (33)	0.23
Duration (years) of MHT use	8.2 (8.1); *n* = 45	9.1 (8); *n* = 13	0.73
The duration (years) of tamoxifen use			
(a) up to the baseline visit		4.2 (1.7)	
(b) from initiation to the baseline visit		6.2 (5.9)	
Breast cancer receptor status			
Positive ER (*n* = 114)	62 (82)	38 (100)	0.004
Positive PR (*n* = 100)	46 (71)	32 (91)	0.017
Positive HER2 (*n* = 23)	1 (6)	1 (14)	NE
TNM^#^ classification			
T classification (*n* = 140)			
T0	2 (2)	0	0.011
Tis	37 (37)	3 (8)	
T1	47 (47)	33 (83)	
T2	10 (10)	4 (10)	
T3	3 (3)	0	
T4	1 (1)	0	
N classification (*n* = 143)			
NX	11 (11)	2 (5)	0.029
N0	80 (78)	29 (71)	
N1	10 (10)	9 (22)	
N2	1 (1)	1 (2)	
Breast cancer treatment			
Surgery	108 (99)	42 (100)	NE
Radiation	56 (51)	23 (55)	0.71
Chemotherapy	17 (16)	7 (17)	0.87

^a^ Characteristic summarized with mean (SD) for continuous features or frequencies and percentages *n* (%) for categorical features. Comparisons of covariates and outcomes between women with and without SERMs were evaluated using two‐sample t tests for continuous features, Wilcoxon rank sum tests for ordinal features, and chi‐square or Fisher exact tests for categorical features. NE = Not evaluated because there was too little variability in the covariate to support statistical testing; BMI = Body mass index; BDI = Beck depression inventory; MHT = Menopausal hormone therapy; ER = estrogen receptor; PR = Progesterone receptor; HER2 = human epidermal growth factor receptor 2; T = Tumor size; N = Regional lymph node status; M = Distant metastasis; T0 = No evidence of primary tumor; Tis = Carcinoma in situ; T1 = Tumor size ≤20 mm in greatest dimension; T2 = Tumor size >20 mm but ≤50 mm in greatest dimension; T3 = Tumor size >50 mm in greatest dimension; T4 = Tumor of any size with direct extension to the chest wall and/or to the skin (ulceration or skin nodules), not including invasion of dermis alone; NX = Regional lymph nodes cannot be assessed; N0 = No regional lymph node metastasis (on imaging, clinical examination or histology); N1 = Metastasis in 1–3 axillary lymph nodes; N2 = Metastasis in 4–9 axillary lymph nodes; All M status are M0 = No clinical or radiographic evidence of distant metastasis.

Associations of tamoxifen with global and domain‐specific z‐scores and odds of MCI are summarized in Table 2. Using linear regression analyses, we found no significant associations between tamoxifen use and global or domain‐specific cognitive decline in univariable models or multivariable‐adjusted models adjusting for age, years of education, BMI, and heart disease. Additionally adjusting for APOE genotype did not appreciably change the results. Similarly, there were no significant associations between tamoxifen use and the odds of MCI. Furthermore, removing the women who underwent chemotherapy did not appreciably change the results.

**TABLE 2:** Comparison of outcomes by tamoxifen exposure among 151 women with breast cancer history

Outcome^a^	No tamoxifen, *n* = 109	Tamoxifen, *n* = 42	Univariable^b^		Adjusted^c^	
			PE (SE)	*p*‐value	PE (SE)	*p*‐value
Memory *z*‐score, (*n* = 149)	−0.3 (1.2)	−0.3 (1.3)	0.1 (0.2)	0.72	0.1 (0.2)	0.58
Executive function *z*‐score, (*n* = 141)	−0.2 (1.2)	−0.5 (1.4)	−0.3 (0.2)	0.16	−0.3 (0.2)	0.16
Language *z*‐score, (*n* = 144)	−0.3 (1.2)	−0.3 (1.2)	0.0 (0.2)	0.86	0.0 (0.2)	0.96
Visuospatial skills *z*‐score, (*n* = 139)	−0.1 (1.1)	−0.3 (1.1)	−0.2 (0.2)	0.41	−0.2 (0.2)	0.37
Global cognition *z*‐score, (*n* = 135)	−0.3 (1.3)	−0.4 (1.2)	−0.1 (0.2)	0.56	−0.1 (0.2)	0.50
Cognitive status						
Unimpaired	92 (84)	33 (79)	**OR (95% CI)** ^b^	***p*‐value**	**OR (95% CI)** ^b^	***p*‐value**
MCI	17 (16)	9 (21)	1.48 (0.60–3.63)	0.40	1.62 (0.62–4.21)	0.32

^a^ Outcomes summarized with mean (SD) or *n* (%).

^b^ Parameter estimates (PEs) and standard errors (SEs) from univariable linear regression models of tamoxifen use to predict *z*‐scores; odds ratio (OR) and 95% confidence interval (CI) from a univariate logistic regression model of tamoxifen use to predict MCI.

^c^ PE and SE from linear regression models of tamoxifen used to predict *z*‐scores adjusted for age, years of education, BMI, and heart disease; OR and 95% CI from a logistic regression model of tamoxifen used to predict MCI adjusted for age. The PEs from linear regression models to predict z‐scores were similar in direction and magnitude after further adjustment for APOE status.

### 3.2 Tamoxifen use and MRI markers

The characteristics of women with MRI data are summarized in Table S1. There were no significant differences in characteristics of women with versus without tamoxifen use. The number of observations included in the analysis for each MRI marker is depicted in Table S2. Of the 151 women with a history of breast cancer; 54 had MRI data (tamoxifen = 16; no tamoxifen = 38). Linear regression analysis revealed no significant association of any brain MRI markers with tamoxifen in women with a history of breast cancer (Aim 1) (Table 3).

**TABLE 3:** Summary of imaging analysis and linear regression results

Imaging^a^	Women with personal history of breast cancer			
	No tamoxifen	Tamoxifen	Linear regression results, Age adjusted	
			PE (SE)	*p*‐value
Hippocampus volume	6.8 (0.67)	6.7 (0.6)	−0.48% (2.64%)	0.86
Temporal lobe cortical thickness	2.81 (0.16)	2.79 (0.13)	−0.69% (1.48%)	0.64

^a^ Imaging data summarized with mean (SD). No tamoxifen group used as the reference. Comparison of hippocampus volume and temporal lobe cortical thickness between tamoxifen and no tamoxifen groups depicted no statistically significant difference. Linear regression analysis did not reveal any statistically significant association between imaging markers and tamoxifen use. PE = parameter estimates (s); SE = standard error. Volume/thickness in regression models are log‐transformed. The estimated coefficients were exponentiated to show percent difference between treatment and referent.

### 3.3 Raloxifene use and cognition

There were 2235 women aged 50 and older who had no prior history of any cancer at their baseline MCSA visit. Of these women, 76 (3%) had a history of raloxifene use, and 2159 (97%) did not, at enrollment in the MSCA. The majority of the women had used raloxifene before they enrolled in the MSCA, but 28 women (36.8%) were using raloxifene at the time of enrollment. Characteristics of participants with and without a history of raloxifene use is depicted in Table 4. Compared with non‐users, those who had taken raloxifene were older (mean age 80 vs. 72, *p* < 0.001), had a significantly lower body mass index (mean BMI 28.4 kg/m^2^ vs. 26.5 kg/m^2^, *p* = 0.009), and more frequently had a history of stroke (9% vs. 3%, *p* = 0.016) or heart disease (37% vs. 26%, *p* = 0.028).

**TABLE 4:** Characteristics of participants without a personal history of breast and any other cancer

	No raloxifene, mean (SD)/*n*(%) *n* = 2159	Raloxifene, mean (SD)/*n*(%) *n* = 76	*p*‐value
**Covariate** ^a^			
Age in years	72 (10)	80 (6)	<0.001
Race (*n* = 2228)			
All others	35 (2)	1 (1)	1.0
White	2117 (98)	75 (99)	
Ethnicity (*n* = 2227)			
Hispanic or Latino	10 (1)	2 (3)	0.06
Not Hispanic or Latino	2141 (99)	74 (97)	
Years of education (*n* = 2231)	14 (2)	14 (3)	0.69
Years of education (*n* = 2231)			
≤12	746 (35)	29 (38)	0.52
>12	1409 (65)	47 (62)	
BMI in kg/m^2^ (*n* = 2187)	28.4 (6.2)	26.5 (5.7)	0.009
BMI in kg/m^2^ (*n* = 2187)			
<18.5	29 (1)	3 (4)	0.008
18.5–24.9	648 (31)	31 (42)	
25.0–29.9	727 (34)	23 (31)	
≥30.0	709 (34)	17 (23)	
Comorbidities			
Diabetes	312 (14)	8 (11)	0.34
Hypertension	1396 (65)	54 (71)	0.25
Stroke	72 (3)	7 (9)	0.016
Heart disease	553 (26)	28 (37)	0.028
BDI II (*n* = 2205)			
<13	1964 (92)	70 (93)	0.72
≥13	166 (8)	5 (7)	
APOE status (*n* = 2117)			
All others	1487 (73)	55 (77)	0.37
Any ε4	559 (27)	16 (23)	
Menopause (*n* = 2220)	2104 (98)	76 (100)	0.40
Age at menopause in years (*n* = 1972)	48 (7)	48 (6)	0.75
MHT use	1177 (55)	47 (62)	0.21
Duration (years) of MHT use	9.4 (7.4); n = 1121	6.2 (6.5); n = 44	0.006
The duration (years) of raloxifene use			
(a)up to the baseline visit		4.7 (3.6)	
(b)from initiation to the baseline visit		4.1 (5)	

^a^ Covariates summarized with mean (SD) or *n* (%). For abbreviations and statistical tests see Table 1.

Associations of raloxifene use with cognitive test scores and odds of MCI are summarized in Table 5. Although univariable regression analysis suggested an association between raloxifene use and worse global and domain‐specific cognitive z‐scores, these associations were attenuated and not significant after multivariable adjustment for age, education, BMI, stroke, and heart disease (Table 5). Additional adjustment for APOE did not appreciably change the results. There was no significant association between raloxifene use and odds of MCI.

**TABLE 5:** Comparison of outcomes by raloxifene exposure among 2235 women without breast and any other cancer

Outcome^a^	No raloxifene *n* = 2159	Raloxifene *n* = 76	Univariable^b^		Adjusted^c^	
			PE (SE)	*p*‐value	PE (SE)	*p*‐value
Memory *z*‐score (*n* = 2202)	−0.2 (1.1)	−0.7 (1.1)	−0.5 (0.1)	<0.001	−0.1 (0.1)	0.48
Executive function *z*‐score (*n* = 2154)	−0.1 (1.1)	−0.8 (1.3)	−0.7 (0.1)	<0.001	−0.2 (0.1)	0.13
Language *z*‐score (*n* = 2161)	−0.2 (1.1)	−0.5 (1.0)	−0.4 (0.1)	0.003	0.0 (0.1)	0.95
Visuospatial skills *z*‐score (*n* = 2151)	−0.1 (1.1)	−0.4 (1.0)	−0.2 (0.1)	0.07	0.1 (0.1)	0.22
Global cognition *z*‐score (*n* = 2097)	−0.2 (1.1)	−0.8 (1.2)	−0.6 (0.1)	<0.001	0.0 (0.1)	0.64
Cognitive status						
Unimpaired	1930 (89)	69 (91)	**OR (95% CI)** ^b^	***p*‐value**	**OR (95% CI)** ^c^	**‐value**
MCI	229 (11)	7 (9)	0.86 (0.39–1.88)	0.70	0.44 (0.18–1.08)	0.07

^a^ Outcomes summarized with mean (SD) or *n* (%).

^b^ Parameter estimates (PEs) and standard errors (SEs) from univariable linear regression models of raloxifene use to predict *z*‐scores; odds ratio (OR) and 95% confidence interval (CI) from a univariable logistic regression model of raloxifene use to predict MCI.

^c^ PE and SE from linear regression models of raloxifene used to predict *z*‐scores adjusted for age, years of education, BMI, stroke, and heart disease; OR and 95% CI from a logistic regression model of raloxifene used to predict MCI adjusted for age, years of education, BMI, stroke, and heart disease. The PEs from linear regression models to predict z‐scores and the OR from a logistic regression model to predict MCI were similar in direction and magnitude after further adjustment for APOE status. MCI=mild cognitive impairment.

### 3.4 Raloxifene use and MRI markers

The characteristics of women with MRI data are summarized in Table S1. Compared with non‐users, those who took raloxifene were older (mean age 78 vs. 72, *p* < 0.001). Of the 2235 women with no personal history of any cancer (Aim 2), 755 had MRI data (raloxifene = 25; no raloxifene = 730) (Table S2). Raloxifene was not significantly associated with any brain MRI markers (Table 6).

**TABLE 6:** Summary of imaging analysis and linear regression results

Imaging^a^	Women with no personal history of breast cancer			
	No raloxifene	Raloxifene	Linear regression results, Age‐adjusted	
			PE (SE)	*p*‐value
Hippocampus volume	7.02 (0.73)	7.00 (0.78)	0.29% (1.69%)	0.86
Temporal lobe cortical thickness	2.87 (0.17)	2.81 (0.13)	−0.16% (1.07%)	0.88

^a^ Imaging data summarized with mean (SD). No raloxifene group used as the reference. Comparison of hippocampus volume and temporal lobe cortical thickness between tamoxifen and no tamoxifen groups depicted no statistically significant difference. Linear regression analysis revealed no statistically significant association between imaging markers and raloxifene use. PE = parameter estimates (s); SE = standard error. Volume/thickness in regression models are log‐transformed. The estimated coefficients were exponentiated to show percent difference between treatment and referent.

## 4 DISCUSSION

In this cross‐sectional study, we investigated the associations of tamoxifen (among women with a history of breast cancer) and raloxifene (among women with no prior history of any cancer) use and global‐ and domain‐specific cognitive performance, odds of MCI, and measures of AD‐related neurodegeneration (hippocampal volume and temporal lobe cortical thickness) among women enrolled in a population‐based study. We did not find significant associations between either tamoxifen or raloxifene use and any outcome.

### 4.1 Tamoxifen

The results of previous studies that have investigated the effect of tamoxifen on cognitive function and risk of AD or dementia have been inconsistent. The present study offers additional support that tamoxifen is not significantly associated with impairment in cognitive function,^33^ and extends previous findings by demonstrating that tamoxifen use was not significantly associated with either global‐ or domain‐specific (memory, attention/executive function, language, visuospatial skills) cognition or odds of MCI. In contrast, some cross‐sectional studies reported an impairment in verbal learning/memory [see meta‐analysis^34^ for more detail], visuospatial ability, attention, executive function, and a global measure of cognitive function in a tamoxifen treatment group compared with a no tamoxifen treatment group. The differences in findings might be attributed to heterogeneity of studies such as variations in age groups between studies, use of wide range of neuropsychological tests, type of control groups (e.g., breast cancer vs non‐cancer controls not using any endocrine therapy), and treatment groups (for example, some studies pooled aromatase inhibitor and tamoxifen users in one treatment group).^34^ For example, a meta‐analysis study reported that some studies that found a significant association between tamoxifen and cognitive performance used non‐breast cancer controls instead of breast cancer controls not using any HMT.^34^ In two studies, women who used either tamoxifen or aromatase inhibitors were pooled into one group.^9^, ^10^ In the present study, we did not collapse tamoxifen users and aromatase users in one treatment group, but we excluded women who used other HMTs such as aromatase inhibitors and raloxifene in Aim 1 of the study.

Our null results between tamoxifen and MCI are consistent with a study that did not find a significant association between tamoxifen and the risk of dementia.^35^ However, findings in the literature are mixed. A historical nationwide case–control study reported that tamoxifen exposure was associated with increased odds of AD in postmenopausal women with breast cancer.^36^ Tamoxifen was also associated with a lower risk of AD history^7^, ^37^ and a lower prevalence of AD.^8^

In the present study, we used MRI markers of neurodegeneration associated with AD, namely hippocampus volume and temporal lobe cortical thickness. Although small hippocampus volume serves as a nonspecific marker of neurodegeneration that could reflect any of the age‐related processes leading to neurodegeneration,^13^ temporal cortical atrophy serves as a MRI marker for risk of cognitive decline associated with AD and related disorders particularly LATE disease.^12^, ^38^ To our knowledge, imaging studies that examined the association of SERMs with structural brain changes are limited. We did not find significant associations between tamoxifen use and any brain MRI markers. In contrast to our study, a modest difference in hippocampal volume between a tamoxifen‐treated and no treatment group (women with no breast cancer who were not taking estrogen or tamoxifen) was reported after exclusion of an outlier.^11^ The different outcomes between these two studies might be attributed to differences in demographic data and comparison groups in both studies, among others. In our study, we included the women with breast cancer who were using tamoxifen but not aromatase inhibitors into our analysis.

### 4.2 Raloxifene

Our results are consistent with findings of several previous studies and extend those results to suggest no significant associations between raloxifene and multiple cognitive domains including memory, executive function, language, visuospatial skills, global cognition, and odds of MCI in postmenopausal women in multivariable analyses.^7^, ^39^, ^40^, ^41^, ^42^ Our findings are also in line with longitudinal studies, including randomized clinical trials assessing the 1‐ and 3‐year effects of raloxifene on cognition.^39^, ^40^, ^43^, ^44^ In contrast to our study and earlier longitudinal studies, a study reported a slight increase in verbal memory performance in women with osteoporosis who used raloxifene (120 mg/day) at twice the typical clinical dose (60 mg/day) compared with placebo at the end of 3 months of treatment.^39^ Few studies have investigated the association of raloxifene exposure with a risk of MCI.^45^ We did not find a significant association in the current study. Our results are in line with a retrospective study that reported no association between raloxifene use and risk of AD or other types of dementia in postmenopausal women with a personal history of breast cancer.^7^

The interpretation, and comparison, of our results with previous studies need careful consideration due to differences in the design of the studies and methodological limitations.^46^ In Aim 1 of this study, we selected both tamoxifen and no tamoxifen groups from women with a personal history of breast cancer to consider the mental and physical effects of cancer diagnosis and cancer therapy (e.g., chemotherapy, radiotherapy, and surgery) on cognitive performance and odds of MCI. However, there are variations in the existing literature regarding selection of comparison groups. For example, women with no personal history of breast cancer were sometimes selected as a comparison group when comparing those who used HMT (SERMs and aromatase inhibitors). This could exacerbate any differences between groups. Inconsistencies in the effects of tamoxifen or raloxifene on cognitive performance might also be due to the heterogeneity in the design of experiments, age groups, menopausal status, dose of SERMs, treatment/therapy follow‐up periods (for longitudinal studies), and neuropsychological cognitive performance batteries.^46^ In our study, characteristics of participants did not differ between SERMs (tamoxifen or raloxifene) users and untreated groups. In addition, some previous studies did not distinguish between tamoxifen and aromatase inhibitors, but instead evaluated the effect of pooled treatment groups (e.g., tamoxifen + aromatase inhibitors) on cognitive performance.^9^, ^10^ The ideal approach would be to investigate the effect of these treatments separately because the mechanisms of tamoxifen and aromatase inhibitors are different. Aromatase inhibitor blocks activity of aromatase, which converts androgens to estrogens, and tamoxifen modulates estrogen receptors. Because the effect of aromatase inhibitor and tamoxifen on cognitive function may differ,^47^ we only evaluated the effect of tamoxifen on cognitive performance in women with a personal breast cancer history. This information may be helpful for those with breast cancer who need to choose an optimum hormone modifying treatment.

Strengths of the study include the population‐based sampling and confirmation of cancer history and use of tamoxifen and raloxifene through medical record abstraction. Furthermore, cognitive performance was evaluated objectively using well‐established tests compared to self‐reports. Limited sample size was a potential limitation of this study. In particular, the number of participants who were treated with tamoxifen and raloxifene and had MRI were relatively small. Another limitation is that the population consists of women who are primarily of European descent and the results may not be generalizable to more racially and ethnically diverse populations or to men who have taken SERMs. Some studies suggest that the duration of HMT does not affect cognitive performance,^48^, ^49^ whereas another study reported that being a current HMT user, but not a past user, may adversely affect cognition.^50^ In our study, the sample size was not sufficient to a do a subgroup analysis to evaluate how duration of use, or current versus past use, of SERMs may affect cognition. In this study, most women were prescribed the typical clinical dose for raloxifene and tamoxifen. Therefore, the modifying effect of dose on associations investigated in this study was not examined. Lastly, it is possible that the current results were biased to the null among women with a breast cancer history because the no tamoxifen arm started out with worse cognitive reserve and thus did not receive the standard of care. Normally, all patients with ER + tumors should be offered HMTs. Thus, the many women in our study who did not receive tamoxifen despite ER+ status suggest that they may not be an average group, and any cognitive impact of tamoxifen may not have been observed.

Studying associations of SERMs exposure with MCI and global or domain‐specific cognition is important to evaluate the potential risk or benefits of SERMs on cognition. Although use of SERMs may decrease the risk of breast cancer or osteoporosis, women who are at risk of developing breast cancer or osteoporosis may hesitate to use these medications if they have doubts about the effects of these medications on cognitive function and the risk of MCI. Because healthy cognitive function is an important part of psychological well‐being, additional studies are needed to evaluate the long‐term effects of these medications on the risk of MCI, cognitive function, and neuroimaging markers of neurodegeneration.

## AUTHOR CONTRIBUTION

Conceptualization: FK and MMM; Study design: FK, CML, JEO, FJC, and KJR; Data analysis: CML, AMC, NT, and TGL; Funding acquisition: MMM, RCP, CRJ, and KK; Writing first draft: FK. All authors participated in the editing and reviewing of the manuscript and approved the final submitted version.

## FUNDING INFORMATION

Funding for this manuscript was provided by the grants from the National Institute on Aging/National Institutes of Health (U54 AG44170, RF1 AG55151, RF1 AG57547 and U01 AG006786). In addition, this study used the resources of the REP medical records‐linkage system, which is supported by the National Institute on Aging (NIA; AG 058738), by the Mayo Clinic Research Committee, and by fees paid annually by REP users.

## CONFLICT OF INTEREST

Dr Jack Jr serves on an independent data monitoring board for Roche, has served as a speaker for Eisai, and consulted for Biogen, but he receives no personal compensation from any commercial entity. He receives research support from NIH and the Alexander Family Alzheimer's Disease Research Professorship of the Mayo Clinic. Dr Petersen received consulting fees from Hoffman‐La Roche, Inc, Merck, Inc, Genentech, Inc, Biogen, Inc, GE Healthcare and Eisai, Inc. Dr Mielke has served as a consultant for Biogen, Brain Protection Company, and LabCorp. All other authors declare no conflicts of interest.

## Supporting information


Table S1

## Data Availability

Data is available upon reasonable request (https://www.mayo.edu/research/centers‐programs/alzheimers‐disease‐research‐center/research‐activities/mayo‐clinic‐study‐aging/for‐researchers/data‐sharing‐resources).
