# Supplementary material for: Association of raloxifene and tamoxifen therapy with cognitive performance, odds of mild cognitive impairment, and brain MRI markers of neurodegeneration
Source: Cancer Med. 2022 Aug 30;12(3):2805–17. doi: 10.1002/cam4.5175 (PMC9939086; doi:10.1002/cam4.5175)
Supplement: Supplementary file 1 — Table S1 [file CAM4-12-2805-s001.docx]

**Supplementary Table 1:** Characteristics of participants with MRI data

|  | Women with history of breast cancer (n= 54) | | | Women without history of breast cancer (n= 755) | | |
| --- | --- | --- | --- | --- | --- | --- |
|  | No tamoxifen  (n= 38) | Tamoxifen  (n= 16) | P-value | No raloxifene  (n= 730) | Raloxifene  (n=25) | P-value |
| APOE ε4 carrier, n (%) | 12 (32%) | 7 (44%) | 0.54 | 203 (28%) | 4 (16%) | 0.26 |
| Education, yr | 13 (2) | 14 (3) | 0.29 | 14 (3) | 14 (2) | 0.65 |
| Age at MRI, yr | 78 (7) | 78 (7) | 0.90 | 72 (10) | 78 (6) | <0.001 |
| CDR Sum of boxes | 0.1 (0.3) | 0.2 (0.5) | 0.44 | 0.1 (0.5) | 0.1 (0.3) | 0.47 |
| MMSE | 28 (2) | 26 (3) | 0.14 | 28 (2) | 28 (1) | 0.91 |

For continuous variables, p-values are from Student's t-test. For categorical variables, p-values are from Fisher's Exact Test. Data shown are mean (SD) listed for the continuous variables or n (%) for the categorical variables. MMSE = Mini-Mental State Examination Score; n = number; MRI= magnetic resonance imaging.

**Supplementary Table 2:** Number of observations included in analysis for brain MRI markers

| MRI | Women with history of breast cancer | | | Women without history of breast cancer | | |
| --- | --- | --- | --- | --- | --- | --- |
|  | Total number | No tamoxifen | Tamoxifen | Total | No raloxifene | Raloxifene |
| Hippocampus volume | 54 | 38 | 16 | 755 | 730 | 25 |
| AD signature atrophy | 51 | 36 | 15 | 723 | 701 | 22 |

MRI= magnetic resonance imaging.
